# PREDICT: a new UK prognostic model that predicts survival following surgery for invasive breast cancer

**DOI:** 10.1186/bcr2464

**Published:** 2010-01-06

**Authors:** Gordon C Wishart, Elizabeth M Azzato, David C Greenberg, Jem Rashbass, Olive Kearins, Gill Lawrence, Carlos Caldas, Paul DP Pharoah

**Affiliations:** 1 Cambridge Breast Unit, Addenbrooke's Hospital, Hills Road, Cambridge, CB2 2QQ, UK; 2 Strangeways Research Laboratory, Department of Oncology, University of Cambridge, Worts Causeway, Cambridge CB1 8RN, UK; 3 Genetic Epidemiology Branch, Division of Cancer Epidemiology and Genetics, National Cancer Institute, Bethesda, MD, USA; 4 Eastern Cancer Registration and Information Centre (ECRIC), Unit C, Magog Court, Shelford Bottom, Hinton Way, Cambridge CB22 3AD, UK; 5 West Midlands Cancer Intelligence Unit, Public Health Building, The University of Birmingham, Birmingham, B15 2TT, UK; 6 Department of Oncology, University of Cambridge, and Functional Breast Cancer Genomics Laboratory, Cancer Research UK Cambridge Research Institute, Li Ka-Shing Centre, Robinson Way, Cambridge CB2 0RE, UK; 7 National Institute of Health Research (NIHR) Cambridge Biomedical Research Centre, Addenbrooke's Hospital, Hills Road, Cambridge, CB2 2QQ, UK

## Abstract

**Introduction:**

The aim of this study was to develop and validate a prognostication model to predict overall and breast cancer specific survival for women treated for early breast cancer in the UK.

**Methods:**

Using the Eastern Cancer Registration and Information Centre (ECRIC) dataset, information was collated for 5,694 women who had surgery for invasive breast cancer in East Anglia from 1999 to 2003. Breast cancer mortality models for oestrogen receptor (ER) positive and ER negative tumours were derived from these data using Cox proportional hazards, adjusting for prognostic factors and mode of cancer detection (symptomatic versus screen-detected). An external dataset of 5,468 patients from the West Midlands Cancer Intelligence Unit (WMCIU) was used for validation.

**Results:**

Differences in overall actual and predicted mortality were <1% at eight years for ECRIC (18.9% vs. 19.0%) and WMCIU (17.5% vs. 18.3%) with area under receiver-operator-characteristic curves (AUC) of 0.81 and 0.79 respectively. Differences in breast cancer specific actual and predicted mortality were <1% at eight years for ECRIC (12.9% vs. 13.5%) and <1.5% at eight years for WMCIU (12.2% vs. 13.6%) with AUC of 0.84 and 0.82 respectively. Model calibration was good for both ER positive and negative models although the ER positive model provided better discrimination (AUC 0.82) than ER negative (AUC 0.75).

**Conclusions:**

We have developed a prognostication model for early breast cancer based on UK cancer registry data that predicts breast cancer survival following surgery for invasive breast cancer and includes mode of detection for the first time. The model is well calibrated, provides a high degree of discrimination and has been validated in a second UK patient cohort.

## Introduction

Accurate prediction of survival is an essential part of the decision making process following surgery for early breast cancer and allows clinicians to determine which patients will benefit from adjuvant therapy. At present these decisions are largely based on known pathological prognostic factors that retain independent significance on multivariate analysis including tumour size, tumour grade and lymph node status in addition to the efficacy of any adjuvant therapy. The predicted treatment benefit can be calculated by applying the relative risk reduction of a particular adjuvant therapy to the breast cancer specific mortality for an individual patient to give an absolute percentage survival benefit for that patient.

The Nottingham Prognostic Index (NPI), a prognostic scoring system based on a large cohort of patients with early breast cancer treated in a single institution, is based on tumour size, grade and lymph node status and when first described divided patients into three groups with significantly different survival [1]. The NPI has been prospectively validated in a second Nottingham dataset [2], as well as in other centres [3], and now allocates patients to one of six prognostic groups [4]. More recently a model has been developed to allow prediction of survival based on individual NPI scores rather than the mean survival of the six groups previously described [5].

Adjuvant! is a web-based prognostication and treatment benefit tool for breast cancer that is now widely used in the UK to help clinicians and patients make decisions about adjuvant therapy. The mortality estimates used in Adjuvant! were based on 10-year observed overall survival (OS) of women aged 36 to 69 who were diagnosed between 1988 and 1992 and recorded in the Surveillance, Epidemiology and End Results (SEER) registry [6]. Breast cancer specific survival (BCSS) without adjuvant therapy was calculated based on estimates of the number of patients likely to have received systemic therapy and the risk reductions outlined in the Early Breast Cancer Trialists' Collaborative Group [7,8]. Although these assumptions have now been validated in a population-based Canadian dataset [9] there has always been some uncertainty about how applicable the Adjuvant! model is to contemporary patients diagnosed and treated in the UK. A recent paper has shown that Adjuvant! overestimated the overall survival by 6% in a UK cohort of 1,065 women with early breast cancer treated in Oxford between 1986 and 1996 [10].

The primary aim of this study therefore was to develop a prognostication model to predict OS from a large cohort of UK women diagnosed in East Anglia from 1999 to 2003 using cancer registration and OS data recorded by the Eastern Cancer Registration and Information Centre (ECRIC). ECRIC provides near complete breast cancer registration for 10 hospitals in East Anglia as well as information on systemic treatment and mode of detection. A secondary aim of this study was to validate the model in a second UK cancer registry dataset to facilitate development of an online prognostication and treatment benefit tool for UK-based patients with early breast cancer.

## Materials and methods

### Study population

The primary analysis was based on data from patients with invasive breast cancer diagnosed in East Anglia, UK between 1999 and 2003 identified by ECRIC. ECRIC covers a catchment area population of approximately 5.5 million people and registers all malignant tumours occurring in people resident in East Anglia at the time of diagnosis. ECRIC also records prospectively demographic, pathologic, staging, general treatment and outcome information. Death certificate flagging through the Office of National Statistics provides the registries with notification of deaths. The lag times for this are a few weeks for cancer deaths and two months to a year for non-cancer deaths. In addition, ECRIC checked vital status by querying the National Health Service Strategic Tracing Service. Vital status was ascertained at the end of June 2008 and all analyses were censored on 31 December 2007 to allow for delay in reporting of vital status. Breast cancer specific mortality was defined as deaths where breast cancer was listed as the cause of death on Parts 1a, 1b, or 1c of the death certificate.

Information obtained from ECRIC included age at diagnosis, number of lymph nodes sampled and number of lymph nodes positive (categorised as 0, 1, 2 to 4, 5 to 9, and 10+ nodes positive), tumour size (categorised as <10 mm, 10 to 19 mm, 20 to 29 mm, 30 to 49 mm, 50+ mm), histological grade (I, II, III), oestrogen receptor (ER) status (positive or negative), mode of detection (screening vs. clinical), information on local therapy (wide local excision, mastectomy, radiotherapy), and type of adjuvant systemic therapy (chemotherapy, endocrine therapy, both). Exact chemotherapy regimens are unknown, but the majority of breast cancer patients in the ECRIC population received first or second generation chemotherapy during this time period. Patients who did not undergo surgery, patients with incomplete local therapy (wide local excision without radiotherapy) and patients with fewer than four nodes excised with a diagnosis of node-negative disease were excluded from the analyses, leaving a study population of 5,694 individuals (Table 1).

**Table 1:** Patient characteristics for model development (Eastern Cancer Registration and Information Centre-ECRIC) and validation (West Midlands Cancer Intelligence Unit-WMCIU) cohorts

	ECRIC		WMCIU	
Total Number of Subjects	5,694		5,468	
				
Total time at risk (years)	31,904		25,917	
Median follow-up (years)*	5.65	(0.04 to 8.00)^†^	4.85	(0.07 to 8.00)^†^
Number of breast cancer deaths	737		668	
Number of other deaths	338		287	
Annual breast cancer mortality rate	0.023	(0.021 to 0.025)^‡^	0.026	(0.024 to 0.028)^‡^
Five-year breast cancer survival rate	0.89	(0.88 to 0.90)^‡^	0.88	(0.87 to 0.89)^‡^
Median age at diagnosis, years	58	(23 to 95)^†^	58	(22 to 93)^†^

	Number		Number	
Age, years				
<35	111	2	108	2
35 to 49	1,172	21	1,195	22
50 to 64	2,630	46	2,393	44
65 to 74	1,124	20	1,101	20
75+	657	12	671	12
Nodal status				
0	3,532	62	3,184	58
1	741	13	746	14
2 to 4	806	14	792	14
5 to 9	380	7	451	8
10+	235	4	295	5
Tumour size, mm				
<10	625	11	485	9
10 to 19	2,310	41	2,136	39
20 to 29	1,627	29	1,566	29
30 to 49	845	15	923	17
50+	287	5	358	7
Grade				
I	1,005	18	1,017	19
II	2,927	51	2,442	45
III	1,762	31	2,009	37
Oestrogen Receptor (ER) Status				
ER negative	991	17	1,116	20
ER positive	4,703	83	4,352	80
Adjuvant therapy				
Chemotherapy	1,905	33	2,121	39
Endocrine therapy	4,268	75	2,406	44
Combined chemoendocrine	1,122	20	579	11
Screen detected				
Yes	1,621	28	1,256	23
No	4,073	72	4,212	77

* Follow-up censored at eight years

^† ^Range of variable

^‡ ^95 CI

An independent validation dataset was comprised of women diagnosed with invasive breast cancer between 1999 and 2003 within the boundaries of the West Midlands Cancer Intelligence Unit (WMCIU). The geographic area served by WMCIU has a population of approximately 5.3 million individuals. Identical patient demographic information and study endpoints were retrieved from the WMCIU cancer registration database, with the same exclusions applied as for the ECRIC dataset. The total validation study population included 5,468 individuals (Table 1). As this was a large population-based study, with full anonymisation of all data, informed consent and ethical approval was not sought.

### Prognostic model parameters

Breast cancer specific mortality and mortality from other causes (competing mortality) were modelled separately. For breast cancer specific mortality, a Cox proportional hazards model was used to estimate the hazard ratio associated with each prognostic factor. As the effect of ER status varies over time [11] ER negative and ER positive tumours were modelled separately. Nodal status, tumour grade and tumour size were modelled both as categorical variables and as ordinal variables. The models with ordinal variables fit the data better, and so these were chosen for the final models. Chemotherapy, endocrine therapy, and tumour detection by screening were treated as simple indicator variables. For the purposes of this study, screen-detected cancers were those discovered by screening mammography in the NHS Breast Screening Programme which at the time offered three-yearly mammography to women aged 50 to 64. In an exploratory analysis, age at diagnosis was included as a categorical variable in five age groups (<40, 40 to 49, 50 to 59, 60 to 69 and 70+) but these were not found to be significantly associated with breast specific mortality (data not shown) and age was excluded from subsequent models.

Competing mortality was modelled separately and adjusted for age at diagnosis. Exploration of the age specific beta-coefficients suggested that the effect varied exponentially with age; the best fit model was age to the power of 2.38.

### Model discrimination and calibration

We used the baseline survivor function from the ER negative and ER positive Cox proportional hazards models for breast cancer specific survival adjusted for the other prognostic factors to estimate the predicted number of deaths from breast cancer. Deaths from other causes were estimated from the baseline survivor function for competing mortality after adjusting for age. The total number of deaths at Years 5 and 8 after diagnosis was estimated by summing the breast-specific and competing mortality. Observed and predicted deaths were compared using a standard Chi-squared test. Model discrimination was evaluated by calculating the area under the receiver-operator-characteristic (ROC) curve (AUC) calculated for breast cancer specific and overall deaths at Year 8 past diagnosis. The ROC curve plots sensitivity against 1-specificity at different predicted risk thresholds. Model calibration was assessed using a simplified goodness-of-fit (GOF) method for the Cox proportional hazards model proposed by May and Hosmer [12] in which observed and model-based estimated deaths at Year 8 after diagnosis within deciles of risk score were compared. This provides a goodness of fit Chi-square test. As the baseline hazards and prognostic variable coefficients differed for ER positive and ER negative models, separate GOF tests were carried out for these models. In subgroup analyses, where numbers within deciles of risk score were small, quartiles of risk scores were used. Person-years lost were calculated by taking the area under the cumulative risk curve. Analyses were performed using STATA, version 9.2 (StataCorp, College Station, TX, USA).

## Results

### Initial model fit

The ECRIC data set was used to derive the primary prognostic models for breast specific and competing mortality. Beta-coefficients and standard errors for each prognostic factor in both the ER negative and ER positive models are provided in Table 2. The estimated relative hazard associated with treatment with adjuvant hormone therapy was smaller than the published estimate based on randomised clinical trials [7,8] in women with ER positive tumours, and was associated with a poorer prognosis in women with ER negative tumours where no effect is expected based on clinical trial data. These differences are likely to represent bias due to clinical selection or patient non-compliance in the observational data.

**Table 2:** Hazard ratios (95% CI) and model coefficients (standard errors) for prognostic factors included in the development models

			ER Positive Model				ER Negative Model	
**Prognostic Factor**	**Hazard ratio**	**95% CI**	**Coefficient**	**SE**	**Hazard ratio**	**95% CI**	**Coefficient**	**SE**
Number Positive Nodes*								
(0, 1, 2 to 4, 5 to 9, 10+)	1.75	1.62 to 1.89	0.56	0.04	1.55	1.44 to 1.68	0.44	0.04
Tumour Size, mm*								
(<10, 10 to 19, 20 to 29, 30 to 49, 50+)	1.43	1.30 to 1.58	0.36	0.05	1.45	1.29 to 1.63	0.37	0.06
Tumour Grade*	1.43	1.30 to 1.58	0.36	0.05	1.45	1.29 to 1.63	0.37	0.06
(Low, Intermediate, High)	1.43	1.30 to 1.58	0.36	0.05	1.45	1.29 to 1.63	0.37	0.06
Detection by Screening	0.70	0.53 to 0.92	-0.36	0.14	0.86	0.56 to 1.32	-0.15	0.22
Chemotherapy	0.73	0.60 to 0.89	-0.31	0.1	0.82	0.62 to 1.08	-0.2	0.14
Hormone therapy	0.95	0.74 to 1.23	-0.05	0.13	1.43	1.09 to 1.89	0.36	0.14

* modeled as ordinal continuous

As expected, this model was well calibrated. The model tended to over-predict mortality, but the difference between actual and predicted deaths was less than one percent at five and eight years after diagnosis (14.8 vs. 15.6 percent and 18.9 vs. 19.0 percent, respectively), differences that were not statistically significant (*P *= 0.10 and 0.83 respectively). There were 31,904 person-years of follow-up compared to 31,662 predicted. Model discrimination was also good - the calculated area under the ROC curve (AUC) for the overall model was 0.81 (SE 0.0074) (Table 3). Similarly, breast cancer specific actual and predicted mortality were within one percent at Years 5 and 8 past diagnosis (10.6 vs. 11.0 percent, *P *= 0.28 and 12.9 vs. 13.5 percent, *P *= 0.26, respectively; AUC = 0.84, SE = 0.008) (Additional file 1, Table S2). The ER positive and ER negative prognostic models were also well-calibrated overall and for all subgroups, and the goodness of fit tests suggest that the models fit well across different risk categories. The ER positive model provided better discrimination (AUC = 0.82, SE = 0.0111) than the ER negative model (AUC = 0.75, SE = 0.0171).

**Table 3:** Overall actual and predicted mortality in Eastern Cancer Registration and Information Centre cohort

			Year 5 deaths*			Year 8 deaths*				
					
Group	N	%	A	P^†^	Mortality Difference	A	P^†^	Mortality Difference	AUC	SE
Total	5,694	100.00	841	890	0.86	1,075	1,082	0.12	0.81	0.007
										
Age, years										
<35	111	1.95	28	23	4.5	31	27	3.6	0.83	0.044
35 to 49	1,172	20.58	150	171	1.79	187	209	1.88	0.81	0.017
50 to 64	2,630	46.19	270	289	0.72	354	359	0.19	0.80	0.013
65 to 74	1,124	19.74	176	191	1.33	227	233	0.53	0.79	0.019
75+	657	11.54	217	216	0.15	276	254	3.35	0.68	0.021
Nodal status										
Negative	3,532	62.03	297	350	1.5	408	433	0.71	0.76	0.013
Positive	2,162	37.97	544	541	0.14	667	649	0.83	0.80	0.010
Tumour size, mm										
<10	625	10.98	30	39	1.44	41	49	1.28	0.75	0.038
10 to 19	2,310	40.57	194	222	1.21	267	280	0.56	0.75	0.017
20 to 29	1,627	28.57	277	283	0.37	363	347	0.98	0.79	0.013
30 to 49	845	14.84	215	226	1.3	259	270	1.3	0.76	0.018
50+	287	5.04	125	119	2.09	145	136	3.14	0.82	0.024
Grade										
I	1,005	17.65	38	64	2.59	55	82	2.69	0.76	0.035
II	2,927	51.40	331	357	0.89	455	445	0.34	0.77	0.013
III	1,762	30.94	472	470	0.11	565	555	0.57	0.77	0.012
Oestrogen Receptor (ER) Status										
Negative	991	17.40	321	318	0.3	364	353	1.11	0.77	0.016
Positive	4,703	82.60	520	572	1.11	711	729	0.38	0.80	0.009

* Number of deaths after censoring follow up at five and eight years after diagnosis.

† Predicted number of deaths rounded to nearest whole number

### Validation

The WMCIU study population of 5,468 individuals was used for independent prognostic model validation. Overall the model was well calibrated. The tendency to over-predict mortality was slightly worse than with the ECRIC data but the difference between actual and predicted mortality was still small (<2 percent) at five years past diagnosis (15.8 vs. 17.4 percent, *P *= 0.004) and less than one percent at Year 8 past diagnosis (17.5 vs. 18.4 percent, *P *= 0.11) (Table 4). There were 25,917 person-years of follow-up compared to 25,809 person-years predicted. The overall model AUC was calculated as 0.79 (SE = 0.0079). Breast cancer specific actual and predicted mortality were within two percent at Years 5 and 8 past diagnosis (11.0 vs. 12.6 percent and 12.2 vs. 13.6 percent, respectively; AUC = 0.82, SE = 0.0083) (Additional file 1, Table S1).

**Table 4:** Overall actual and predicted mortality in West Midland Cancer Intelligence Unit (WMCIU) cohort

			Year 5 deaths*			Year 8 deaths*				
					
Group	N	%	A	P^†^	Mortality Difference	A	P^†^	Mortality Difference	AUC	SE
Total	5,468	100	862	950	1.61	955	1006	0.93	0.79	0.008
										
Age, years										
<35	108	1.98	21	24	2.78	28	26	1.85	0.70	0.057
35 to 49	1,195	21.85	153	185	2.68	175	201	2.18	0.79	0.018
50 to 64	2,393	43.76	279	311	1.34	310	334	1	0.80	0.013
65 to 74	1,101	20.14	198	203	0.45	218	217	0.09	0.76	0.018
75+	671	12.27	211	216	0.75	224	228	0.6	0.72	0.021
Nodal status										
Negative	3,184	58.23	265	333	2.14	301	357	1.76	0.74	0.015
Positive	2,284	41.77	597	606	0.39	654	648	0.26	0.75	0.011
Tumour size, mm										
<10	485	8.87	27	32	1.03	29	34	1.03	0.82	0.040
10 to 19	2,136	39.06	173	216	2.01	196	233	1.73	0.76	0.018
20 to 29	1,566	28.64	259	274	0.96	286	295	0.57	0.71	0.017
30 to 49	923	16.88	257	258	0.11	272	276	0.43	0.72	0.018
50+	358	6.55	146	160	3.91	156	168	3.35	0.72	0.027
Grade										
I	1,017	18.6	66	67	0.1	75	72	0.29	0.79	0.029
II	2,442	44.66	314	318	0.16	359	344	0.61	0.77	0.013
III	2,009	36.74	482	554	3.58	521	589	3.38	0.75	0.012
Oestrogen Receptor (ER) Status										
Negative	1,116	20.41	317	364	4.21	341	380	3.49	0.76	0.016
Positive	4,352	79.59	545	575	0.69	614	625	0.25	0.78	0.010

* Number of deaths after censoring follow up at five and eight years after diagnosis.

† Predicted number of deaths rounded to nearest whole number

Overall, the ER positive and ER negative prognostic models were well-calibrated, although both models predict more breast cancer deaths than observed. The overestimation was slightly greater for the ER negative model than the ER positive model. In ER negative disease, the Year 8 actual breast cancer mortality rate was 25.0 percent compared to 30.6 percent predicted; for ER positive tumours, Year 8 actual and predicted breast cancer mortality were within one percent (8.9 vs. 9.2 percent). Overall model fit was good (GOF *P*-values > 0.05), although the fit was less good for some sub-groups. Specifically, for ER positive disease, the fit was not so good in women aged <35 years (GOF *P *= 0.01) and 35 to 49-year-old age category (*P *= 0.045). For ER negative disease, the model fit in node negative disease (GOF *P *= 0.03), 30 to 49 mm tumours size category (GOF *P *= 0.02) and high grade tumours (GOF *P *= 0.001) was not so good (Additional file 1, Table S2).

**Figure 1:**
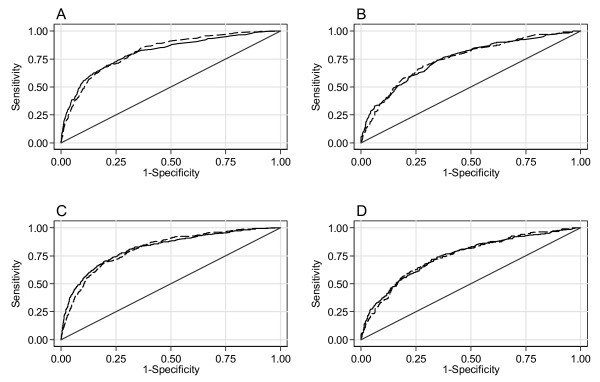
**Receiver operator characteristic curves for breast cancer specific mortality by Oestrogen Receptor status in Eastern Cancer Registration and Information Centre and West Midlands Cancer Intelligence Unit cohorts**. **A) **ER positive at five years, **B) **ER negative at five years, **C) **ER positive at eight years, **D) **ER negative at eight years. Solid line ECRIC data; dashed line WMCIU data.

Model discrimination was also good, again being somewhat better for the ER positive model (AUC = 0.81, SE = 0.0111) than the ER negative model (AUC = 0.75, SE = 0.0169). There were no significant differences between the ROC curves generated with the ECRIC and WMCIU data (ER positive χ^2 ^= 0.17, *P *= 0.68, ER negative χ^2 ^= 0.00, *P *= 0.95) (Figure 1).

We also explored the overall and breast cancer specific mortality within T1N0 and T2N0 good prognosis subgroups where decisions regarding adjuvant therapy can be difficult and challenging (Additional file 1, Table S3). In the WMCIU population, 1,931 individuals were diagnosed with T1N0 tumours, while 1,182 individuals were diagnosed with T2N0 tumours. For T1N0 tumours, actual and predicted five- and eight-year overall mortality rates were within 2.1 percent (5.5 vs. 7.6 percent and 6.1 vs. 8.2 percent, respectively); actual and predicted five- and eight-year breast cancer specific mortality rates were within one percent (2.4 vs. 3.3 percent and 2.8 vs. 3.6 percent, respectively). For T2N0 tumours, actual and predicted five- and eight-year overall mortality was within 2.5 percent (11.7 vs. 14.1 percent and 13.5 vs. 15.2 percent, respectively); actual and predicted five- and eight-year breast cancer specific mortality was within one percent (7.9 vs. 8.7 percent and 9.1 vs. 9.4 percent, respectively).

### Summary comparison of overview vs. model-derived therapy benefit estimates

Given the difference in the estimates of the effects of hormone therapy from the ECRIC dataset compared to published clinical trial data, we also fit models (constrained models) with the relative hazard of hormone therapy constrained to the published estimate from the 1998 overviews (relative hazard 0.68 for ER positive tumours). Under this constrained model, the coefficient estimates for the other prognostic factor coefficients were similar to the original, data-driven model (Additional file 1, Table S4). Performance of the constrained model was slightly poorer in the ECRIC data than the full data driven model (Table 5), but the difference between actual and predicted mortality at eight years and between actual and predicted person-years of follow-up was still small. In the WMCIU validation dataset, the constrained model performed better than the full, data-driven model at predicting eight-year mortality and person-years of follow-up.

**Table 5:** Comparison of mortality and person-years lost for data-derived and constrained models in development and validation cohorts

			All cause mortality			Breast specific mortality			Person-Years Lost		
ECRIC Model:	Hormone coefficient	Chemotherapy coefficient	A	P	ROC	A	P	ROC	Total Possible PY^‡^	A	P
Full	Model	Model	1,075	1,082	0.81	737	768	0.84	35,003	3,099	3,341
Full	Overview*	Overview	1,075	984	0.81	737	660	0.83	35,003	3,099	3,030
Constrained	Model^†^	Model	1,075	980	0.81	737	656	0.83	35,003	3,099	3,061
Constrained	Overview	Overview	1,075	990	0.81	737	667	0.83	35,003	3,099	3,089
											
**WMCIU** Model:											
Full	Model	Model	955	1,006	0.79	668	743	0.82	28,322	2,405	2,513
Full	Overview	Overview	955	956	0.79	668	690	0.81	28,322	2,405	2,376
Constrained	Model^†^	Model	955	952	0.78	668	685	0.81	28,322	2,405	2,406
Constrained	Overview	Overview	955	965	0.78	668	699	0.81	28,322	2,405	2,436

*Application of overview estimates is performed by setting the model-derived therapy coefficient to zero. Overview-derived therapy risk reductions (first generation) are then applied in the life table analysis. The risk reductions are only applied to individuals who actually received therapy. No Overview hormone therapy benefit was applied to individuals with ER- tumors.

^†^Under the constrained model, the model coefficient for adjuvant hormone therapy is the same as the Overview estimate.

^‡^Follow-up was censored at eight years

Finally, we tested the performance of models using the data derived coefficients for grade, node status, tumour size and mode of detection from the full and constrained models with the benefit estimates from the 1998 overviews (Table 5). First generation chemotherapy benefit estimates were applied in all these analyses. Again the models performed slightly poorer than the full, data-driven model in the ECRIC dataset, but somewhat better in the WMCIU validation dataset.

## Discussion

We have developed a prognostication model for early breast cancer based on data collated from a large number of patients within a single UK cancer registry. The model was validated using data from a second UK registry. As both model and validation datasets contain over 5,000 patients this model is likely to be predictive of overall survival for all women diagnosed with early breast cancer in the UK. The model was well calibrated and provides a high degree of discrimination across different prognostic groups. A particular strength of this project was the ability to access breast cancer specific mortality from ECRIC, based on death certificate reporting rather than being estimated from population data.

Accurate prediction of survival, and subsequent calculation of treatment benefit, has become increasingly sophisticated in the management of early breast cancer in the UK. Although the introduction of the NPI allowed risk stratification into five then six [4] prognostic groups, the original models provided survival estimates based on the average survival for each individual group. Furthermore, the model was based on treatment from a single institution where individual treatment bias may have an effect on overall survival. Despite this potential shortcoming, the NPI has been successfully validated in external datasets [3] and has now been further developed to include more *individual *survival prediction based on individual rather than group NPI scores [5].

The publication of the Adjuvant! prognostication and treatment benefit tool in 2001 led to widespread and early adoption in the UK. The web-based system allowed free access and was recognised as being user friendly for both clinicians as well as patients with breast cancer. Adjuvant! was seen to provide several advantages over and above the NPI including individual survival predictions and calculation of potential treatment benefits for that patient. The use of coloured bar charts to display this information facilitated the often difficult discussions surrounding systemic adjuvant therapies with patients and allowed the development of treatment thresholds for chemotherapy in individual breast units.

The Adjuvant! model is based on population data collected by the Surveillance, Epidemiology and End Results (SEER) registry [6]. Breast cancer specific survival (BCSS) without adjuvant therapy was calculated based on estimates of the number of patients likely to have received systemic therapy and the risk reductions outlined in the Early Breast Cancer Trialists' Collaborative Group [7,8]. In contrast, systemic therapy was recorded for all patients used to generate this model, as well as breast cancer specific mortality. Breast cancer registration is close to 100 percent for both SEER and ECRIC data across specific geographic regions. This may limit their generalisability, but the good performance of the model based on ECRIC data in an independent dataset from a different region of the UK and validation of Adjuvant! using data from a population registry from Canada [9] suggests that this is not likely to be a significant problem.

A key aim that underpinned development of this model was to develop a prognostication and treatment benefit tool that benefited from the many attributes of the Adjuvant! model but which was specifically tailored to the UK population. UK cancer registries have near complete prospective data collection on breast cancer registration, pathological features, treatment and death notification. The ECRIC data used in this study included all female breast cancer cases that were treated surgically and were fully characterised for mode of detection, tumour size and grade, lymph node and ER status and details of adjuvant therapy. ECRIC collects data from more than 10 hospitals in East Anglia including only two teaching hospitals with strong research activity. As a result the data collected by ECRIC are likely to be representative of the UK as a whole and reflect *good practise *rather than *best practise *and was an ideal data source on which to base the initial model.

In addition, the success of the NHS Breast Screening Programme in the UK has meant that there has been a shift to better prognostic groups at diagnosis than previously. Two recent papers, have suggested that screen detection confers an additional survival benefit beyond stage shift and reduces the risk of systemic recurrence when compared with symptomatic cancers of a similar stage [13,14]. Although the majority of the survival advantage associated with breast screening can be explained by this shift to an earlier stage at diagnosis, recent evidence suggests that approximately 25 percent of the survival advantage is still unexplained [15]. Introduction of mode of detection (screen-detected versus symptomatic) was therefore a key requirement for this model, as was adjustment of the nodal status groups with creation of a single node positive group. The inclusion of a group with a single positive node will allow these patients to have more accurate survival prediction than previously, as prognosis in Adjuvant! is based on the average of the one to three node positive group.

The model performs well across all prognostic groups in the development (ECRIC) dataset except in patients ≥ 75 years old, where the predicted mortality at Year 8 past diagnosis was less than observed (250 predicted vs. 276 actual deaths). This was also seen in the validation (WMCIU) data. In these data the model also predicted a more favourable outcome than observed for low grade tumours and a less favourable outcome than observed for high grade and ER negative tumours.

A key decision, when considering the application of this model as a predictor of treatment benefit, is whether to use the data-derived coefficients for hormone therapy or chemotherapy or the benefit estimates from published overview data [7,8]. The application of the overview estimates to the full model was a strong predictor of both eight-year mortality and person-years follow-up in the WMCIU validation dataset and has the advantage of allowing regular updates as further overview results are published.

## Conclusions

In conclusion we have developed a prognostication model for early breast cancer based on data from a UK cancer registry that has included mode of detection for the first time. The model is well calibrated, provides a high degree of discrimination and has been validated in a second UK patient cohort. This model, together with application of published relative risk reductions for systemic therapy, will underpin a new web-based prognostication and treatment benefit tool for early breast cancer in the UK.

## Abbreviations

AUC: area under ROC curve; BCSS: breast cancer specific survival; ECRIC: Eastern Cancer Registration and Information Centre; ER: oestrogen receptor; GOF: goodness-of-fit; NPI: Nottingham Prognostic Index; OS: overall survival; ROC: receiver-operator characteristic; SEER: Surveillance, Epidemiology and End Results; WMCIU: West Midland Cancer Intelligence Unit.

## Competing interests

The authors declare that they have no competing interests.

## Authors' contributions

GCW conceived of the project and participated in the design, analysis and writing of the manuscript. EMA participated in the design, statistical analysis and writing of the manuscript. PDPP participated in the design, statistical analysis and writing of the study. DCG participated in the design, data acquisition, analysis and writing of the manuscript. JR participated in the design and writing of the manuscript. OK participated in the data acquisition and writing of the manuscript. GL participated in the data acquisition, analysis and writing of the manuscript. CC participated in the design, analysis and writing of the manuscript.

## Supplementary Material

Additional file 1 **Tables S1-5**. Table S1 contains actual and predicted breast cancer mortality in the Eastern Cancer Registration and Information Centre (ECRIC) cohort. Table S2 contains actual and predicted breast cancer mortality in the West Midland Cancer Intelligence Unit (WMCIU) cohort. Table S3 contains breast cancer specific and overall mortality in patients with good prognosis (T1N0 and T2N0) tumours in the West Midland Cancer Intelligence Unit (WMCIU) cohort. Table S4 contains beta coefficients and standard errors for prognostic factors included in the constrained Eastern Cancer Registration and Information Centre (ECRIC) breast cancer prognostic models. Table S5 contains the baseline survival for breast cancer mortality by Oestrogen Receptor (ER) status and competing mortality. Click here for file
