# Supplementary material for: PREDICT: a new UK prognostic model that predicts survival following surgery for invasive breast cancer
Source: Breast Cancer Res. 2010 Jan 6;12(1):R1. doi: 10.1186/bcr2464 (PMC2880419; doi:10.1186/bcr2464)
Supplement: Additional file 1 — Tables S1-5. Table S1 contains actual and predicted breast cancer mortality in the Eastern Cancer Registration and Information Centre (ECRIC) cohort. Table S2 contains actual and predicted breast cancer mortality in the West Midland Cancer Intelligence Unit (WMCIU) cohort. Table S3 contains breast cancer specific and overall mortality in patients with good prognosis (T1N0 and T2N0) tumours in the West Midland Cancer Intelligence Unit (WMCIU) cohort. Table S4 contains beta coefficients and standard errors for prognostic factors included in the constrained Eastern Cancer Registration and Information Centre (ECRIC) breast cancer prognostic models. Table S5 contains the baseline survival for breast cancer mortality by Oestrogen Receptor (ER) status and competing mortality. [file bcr2464-S1.doc]

Table S1. Actual and Predicted breast cancer mortality in Eastern Cancer Registration and Information Centre (ECRIC) cohort by subgroup and Oestrogen Receptor (ER) status.

|  |  |  | **Year 5** | | | **Year 8** | | |  |  |  |  |
| --- | --- | --- | --- | --- | --- | --- | --- | --- | --- | --- | --- | --- |
| **Group** | **N** | **%** | **A** | **P** | **Mort**  **Diff** | **A** | **P** | **Mort**  **Diff** | **GOF**  **Chi-sq** | **GOF**  **p*** | **ROC**  **Area** | **SE** |
| **Total** | 5694 |  | 601 | 628 | 0.47 | 737 | 768 | 0.54 | n/a | n/a | 0.84 | 0.008 |
|  |  |  |  |  |  |  |  |  |  |  |  |  |
| **ER Pos** | 4703 | 100 | 327 | 343 | 0.34 | 436 | 454 | 0.38 | 6.27 | 0.71 | 0.82 | 0.011 |
|  |  |  |  |  |  |  |  |  |  |  |  |  |
| Age, years |  |  |  |  |  |  |  |  |  |  |  |  |
| <35 | 59 | 1 | 10 | 7 | 5.08 | 12 | 9 | 5.08 | 1.98 | 0.58 | 0.85 | 0.053 |
| 35-49 | 908 | 19 | 67 | 80 | 1.43 | 94 | 107 | 1.43 | 4.93 | 0.18 | 0.78 | 0.026 |
| 50-64 | 2220 | 47 | 117 | 122 | 0.23 | 164 | 165 | 0.05 | 3.35 | 0.34 | 0.84 | 0.018 |
| 65-74 | 969 | 21 | 67 | 73 | 0.62 | 86 | 96 | 1.03 | 1.85 | 0.60 | 0.82 | 0.026 |
| 75+ | 547 | 12 | 66 | 61 | 0.91 | 80 | 77 | 0.55 | 0.38 | 0.94 | 0.77 | 0.029 |
| Nodal status |  |  |  |  |  |  |  |  |  |  |  |  |
| Negative | 2984 | 63 | 85 | 88 | 0.10 | 116 | 123 | 0.23 | 1.99 | 0.57 | 0.71 | 0.025 |
| Positive | 1719 | 37 | 242 | 255 | 0.76 | 320 | 332 | 0.70 | 2.85 | 0.42 | 0.79 | 0.014 |
| Tumour size, mm |  |  |  |  |  |  |  |  |  |  |  |  |
| <10 | 564 | 12 | 6 | 9 | 0.53 | 7 | 12 | 0.89 | 3.23 | 0.36 | 0.86 | 0.058 |
| 10-19 | 1997 | 42 | 69 | 68 | 0.05 | 94 | 96 | 0.10 | 2.36 | 0.50 | 0.69 | 0.031 |
| 20-29 | 1292 | 27 | 107 | 107 | 0.00 | 150 | 145 | 0.39 | 0.83 | 0.84 | 0.79 | 0.020 |
| 30-49 | 643 | 14 | 94 | 103 | 1.40 | 119 | 132 | 2.02 | 4.28 | 0.23 | 0.76 | 0.024 |
| 50+ | 207 | 4 | 51 | 56 | 2.42 | 66 | 69 | 1.45 | 3.09 | 0.21^†^ | 0.82 | 0.029 |
| Grade |  |  |  |  |  |  |  |  |  |  |  |  |
| I | 977 | 21 | 18 | 15 | 0.31 | 22 | 21 | 0.10 | 6.45 | 0.09 | 0.72 | 0.053 |
| II | 2689 | 57 | 137 | 152 | 0.56 | 195 | 204 | 0.33 | 3.29 | 0.35 | 0.77 | 0.019 |
| III | 1037 | 22 | 172 | 176 | 0.39 | 219 | 228 | 0.87 | 2.3 | 0.32^†^ | 0.77 | 0.018 |
|  |  |  |  |  |  |  |  |  |  |  |  |  |
| **ER Neg** | 991 | 100 | 274 | 285 | 1.11 | 301 | 314 | 1.31 | 5.81 | 0.76 | 0.75 | 0.017 |
|  |  |  |  |  |  |  |  |  |  |  |  |  |
| Age, years |  |  |  |  |  |  |  |  |  |  |  |  |
| <35 | 52 | 5 | 17 | 15 | 3.85 | 18 | 17 | 1.92 | 4.28 | 0.23 | 0.88 | 0.057 |
| 35-49 | 264 | 27 | 71 | 79 | 3.03 | 77 | 87 | 3.79 | 2.97 | 0.40 | 0.72 | 0.036 |
| 50-64 | 410 | 41 | 95 | 104 | 2.20 | 110 | 116 | 1.46 | 0.62 | 0.89 | 0.76 | 0.027 |
| 65-74 | 155 | 16 | 48 | 47 | 0.65 | 53 | 52 | 0.65 | 1.32 | 0.72 | 0.72 | 0.044 |
| 75+ | 110 | 11 | 43 | 40 | 2.73 | 43 | 43 | 0.00 | 3.75 | 0.29 | 0.73 | 0.051 |
| Nodal status |  |  |  |  |  |  |  |  |  |  |  |  |
| Negative | 548 | 55 | 85 | 92 | 1.28 | 94 | 103 | 1.64 | 2.37 | 0.50 | 0.65 | 0.032 |
| Positive | 443 | 45 | 189 | 193 | 0.90 | 207 | 211 | 0.90 | 2.75 | 0.43 | 0.69 | 0.025 |
| Tumour size, mm |  |  |  |  |  |  |  |  |  |  |  |  |
| <10 | 61 | 6 | 7 | 6 | 1.64 | 9 | 7 | 3.28 | 2.13 | 0.34^†^ | 0.69 | 0.092 |
| 10-19 | 313 | 32 | 51 | 56 | 1.60 | 63 | 64 | 0.32 | 0.89 | 0.83 | 0.71 | 0.040 |
| 20-29 | 335 | 34 | 92 | 92 | 0.00 | 99 | 102 | 0.90 | 2.13 | 0.55 | 0.70 | 0.032 |
| 30-49 | 202 | 20 | 71 | 81 | 4.95 | 76 | 89 | 6.44 | 2.43 | 0.49 | 0.67 | 0.020 |
| 50+ | 80 | 8 | 53 | 51 | 2.50 | 54 | 53 | 1.25 | 0.47 | 0.79^†^ | 0.73 | 0.062 |
| Grade |  |  |  |  |  |  |  |  |  |  |  |  |
| I | 28 | 3 | 0 | 3 | 10.7 | 0 | 4 | 14.29 | 3.64 | 0.16^†^ | n/a | |
| II | 238 | 24 | 63 | 56 | 2.94 | 70 | 63 | 2.94 | 1.63 | 0.65 | 0.74 | 0.038 |
| III | 725 | 73 | 211 | 226 | 2.07 | 231 | 248 | 2.34 | 3.24 | 0.36 | 0.74 | 0.020 |
| Goodness-of-fit (GOF) based on 8 years | |  |  |  |  |  |  |  |  |  |  |  |
| *ER groups based on deciles of risk (9 Degrees Of Freedom (DOF) test), subgroups based on quartiles of risk (3 DOF test) | | | | | | | | | | | | |
| ^†^2 DOF test--none Predicted in one quartile | | | | | | | | | | | | |

Table S2. Actual and predicted breast cancer mortality in West Midlands Cancer Intelligence Unit (WMCIU) cohort by subgroup and Oestrogen Receptor (ER) status.

|  |  |  | **Year 5** | | | **Year 8** | | |  |  |  |  |
| --- | --- | --- | --- | --- | --- | --- | --- | --- | --- | --- | --- | --- |
| **Group** | **N** | **%** | **A** | **P** | **Mort**  **Diff** | **A** | **P** | **Mort**  **Diff** | **GOF**  **Chi-sq** | **GOF**  **p*** | **ROC**  **Area** | **SE** |
| **Total** | 5468 |  | 602 | 691 | 1.63 | 668 | 743 | 1.37 | n/a | n/a | 0.82 | 0.010 |
|  |  |  |  |  |  |  |  |  |  |  |  |  |
| **ER Pos** | 4352 | 100 | 344 | 364 | 0.46 | 389 | 401 | 0.28 | 11.61 | 0.24 | 0.81 | 0.011 |
|  |  |  |  |  |  |  |  |  |  |  |  |  |
| Age, years |  |  |  |  |  |  |  |  |  |  |  |  |
| <35 | 66 | 1.5 | 9 | 9 | 0.00 | 14 | 10 | 6.06 | 10.55 | **0.01** | 0.73 | 0.081 |
| 35-49 | 931 | 21 | 69 | 93 | 2.58 | 84 | 104 | 2.15 | 8.04 | **0.045** | 0.77 | 0.027 |
| 50-64 | 1902 | 44 | 120 | 127 | 0.37 | 134 | 139 | 0.26 | 4.68 | 0.20 | 0.85 | 0.016 |
| 65-74 | 907 | 21 | 85 | 79 | 0.66 | 91 | 86 | 0.55 | 0.77 | 0.86 | 0.83 | 0.023 |
| 75+ | 546 | 13 | 61 | 57 | 0.73 | 66 | 62 | 0.73 | 5.72 | 0.13 | 0.74 | 0.035 |
| Nodal status |  |  |  |  |  |  |  |  |  |  |  |  |
| Negative | 2568 | 59 | 83 | 79 | 0.16 | 97 | 87 | 0.39 | 3.15 | 0.37 | 0.74 | 0.025 |
| Positive | 1784 | 41 | 261 | 285 | 1.35 | 292 | 314 | 1.23 | 2.7 | 0.44 | 0.76 | 0.014 |
| Tumour size, mm |  |  |  |  |  |  |  |  |  |  |  |  |
| <10 | 425 | 9.8 | 7 | 7 | 0.00 | 8 | 7 | 0.24 | 4.7 | 0.20 | 0.74 | 0.119 |
| 10-19 | 1803 | 41 | 58 | 69 | 0.61 | 70 | 77 | 0.39 | 1.5 | 0.68 | 0.79 | 0.029 |
| 20-29 | 1204 | 28 | 107 | 102 | 0.42 | 126 | 114 | 1.00 | 7.17 | 0.07 | 0.72 | 0.025 |
| 30-49 | 673 | 15 | 110 | 111 | 0.10 | 118 | 123 | 0.74 | 4.59 | 0.20 | 0.72 | 0.026 |
| 50+ | 247 | 5.7 | 62 | 75 | 5.26 | 67 | 81 | 5.67 | 2.49 | 0.29^†^ | 0.74 | 0.033 |
| Grade |  |  |  |  |  |  |  |  |  |  |  |  |
| I | 989 | 23 | 19 | 16 | 0.30 | 24 | 18 | 0.61 | 2.31 | 0.51 | 0.77 | 0.053 |
| II | 2242 | 52 | 140 | 146 | 0.27 | 160 | 162 | 0.09 | 2.29 | 0.51 | 0.78 | 0.019 |
| III | 1121 | 26 | 185 | 202 | 1.52 | 205 | 222 | 1.52 | 5.11 | 0.08 | 0.74 | 0.018 |
|  |  |  |  |  |  |  |  |  |  |  |  |  |
| **ER Neg** | 1116 | 100 | 258 | 327 | 6.18 | 279 | 342 | 5.65 | 15.24 | 0.08 | 0.75 | 0.017 |
|  |  |  |  |  |  |  |  |  |  |  |  |  |
| Age, years |  |  |  |  |  |  |  |  |  |  |  |  |
| <35 | 42 | 3.8 | 11 | 15 | 9.52 | 12 | 15 | 7.14 | 1.37 | 0.71 | 0.65 | 0.097 |
| 35-49 | 264 | 24 | 70 | 80 | 3.79 | 74 | 84 | 3.79 | 3.68 | 0.3 | 0.77 | 0.031 |
| 50-64 | 491 | 44 | 94 | 129 | 7.13 | 105 | 135 | 6.11 | 7.14 | 0.07 | 0.75 | 0.028 |
| 65-74 | 194 | 17 | 44 | 57 | 6.70 | 48 | 59 | 5.67 | 4.36 | 0.23 | 0.69 | 0.045 |
| 75+ | 125 | 11 | 39 | 46 | 5.60 | 40 | 48 | 6.40 | 1.51 | 0.68 | 0.75 | 0.046 |
| Nodal status |  |  |  |  |  |  |  |  |  |  |  |  |
| Negative | 616 | 55 | 69 | 101 | 5.19 | 77 | 106 | 4.71 | 9.01 | **0.03** | 0.64 | 0.034 |
| Positive | 500 | 45 | 189 | 226 | 7.40 | 202 | 235 | 6.60 | 7.87 | **0.049** | 0.66 | 0.025 |
| Tumour size, mm |  |  |  |  |  |  |  |  |  |  |  |  |
| <10 | 60 | 5.4 | 5 | 7 | 3.33 | 6 | 7 | 1.67 | 3.4 | 0.33 | 0.83 | 0.110 |
| 10-19 | 333 | 30 | 46 | 54 | 2.40 | 50 | 57 | 2.10 | 1.46 | 0.69 | 0.68 | 0.042 |
| 20-29 | 362 | 32 | 80 | 93 | 3.59 | 89 | 99 | 2.76 | 4.92 | 0.18 | 0.72 | 0.032 |
| 30-49 | 250 | 22 | 77 | 104 | 10.80 | 81 | 108 | 10.80 | 7.39 | **0.02**^†^ | 0.70 | 0.034 |
| 50+ | 111 | 10 | 50 | 69 | 17.10 | 53 | 70 | 15.32 | 5.18 | 0.07^†^ | 0.66 | 0.053 |
| Grade |  |  |  |  |  |  |  |  |  |  |  |  |
| I | 28 | 2.5 | 3 | 3 | 0.00 | 3 | 4 | 3.57 | 3.23 | 0.2^†^ | 0.89 | 0.064 |
| II | 200 | 18 | 45 | 45 | 0.00 | 52 | 48 | 2.00 | 3.7 | 0.3 | 0.74 | 0.039 |
| III | 888 | 80 | 210 | 279 | 7.77 | 224 | 290 | 7.43 | 15.83 | **0.001** | 0.75 | 0.019 |
| Goodness-Of-Fit based on 8 years | |  |  |  |  |  |  |  |  |  |  |  |
| *ER groups based on deciles of risk (9 Degrees Of Freedom (DOF) test), subgroups based on quartiles of risk (3 DOF test) | | | | | | | | | | | | |
| ^†^2 DOF test--none Predicted in one quartile | | | |  |  |  |  |  |  |  |  |  |

Table S3. West Midlands Cancer Intelligence Unit (WMCIU) mortality by T1N0 and T2N0 groups and Oestrogen Receptor (ER) status

|  |  |  | **Breast Cancer Specific Deaths** | | | | | | **Overall Deaths** | | | | | |
| --- | --- | --- | --- | --- | --- | --- | --- | --- | --- | --- | --- | --- | --- | --- |
|  |  |  | **5 year deaths** | | | **8 year deaths** | | | **5 year deaths** | | | **8 year deaths** | | |
|  | N | % | Actual | Predicted | MortDiff | Actual | Predicted | MortDiff | Actual | Predicted | MortDiff | Actual | Predicted | MortDiff |
| **T1N0** | 1931 | 100 | 48 | 64 | 0.83 | 54 | 69 | 0.78 | 106 | 147 | 2.12 | 118 | 158 | 2.07 |
| Age, years |  |  |  |  |  |  |  |  |  |  |  |  |  |  |
| <35 | 19 | 1 | 1 | 1 | 0 | 1 | 1 | 0 | 1 | 1 | 0 | 1 | 1 | 0 |
| 35-49 | 354 | 18 | 8 | 14 | 1.69 | 10 | 15 | 1.41 | 9 | 18 | 2.54 | 12 | 20 | 2.26 |
| 50-64 | 1009 | 52 | 15 | 31 | 1.59 | 18 | 33 | 1.49 | 36 | 56 | 1.98 | 42 | 60 | 1.78 |
| 65-74 | 394 | 20 | 16 | 12 | 1.02 | 17 | 13 | 1.02 | 33 | 38 | 1.27 | 35 | 40 | 1.27 |
| 75+ | 155 | 8 | 8 | 6 | 1.29 | 8 | 7 | 0.65 | 27 | 34 | 4.52 | 28 | 36 | 5.16 |
| Grade |  |  |  |  |  |  |  |  |  |  |  |  |  |  |
| I | 606 | 31 | 3 | 6 | 0.5 | 5 | 6 | 0.17 | 20 | 30 | 1.65 | 23 | 33 | 1.65 |
| II | 894 | 46 | 18 | 23 | 0.56 | 21 | 25 | 0.45 | 47 | 65 | 2.01 | 54 | 70 | 1.79 |
| III | 431 | 22 | 27 | 36 | 2.09 | 28 | 38 | 2.32 | 39 | 41 | 0.46 | 41 | 55 | 3.25 |
| ER Status |  |  |  |  |  |  |  |  |  |  |  |  |  |  |
| Negative | 275 | 14 | 22 | 31 | 3.27 | 24 | 32 | 2.91 | 33 | 41 | 2.91 | 35 | 43 | 2.91 |
| Positive | 1656 | 86 | 26 | 33 | 0.42 | 30 | 37 | 0.42 | 73 | 106 | 1.99 | 83 | 114 | 1.87 |
|  |  |  |  |  |  |  |  |  |  |  |  |  |  |  |
| **T2N0** | 1182 | 100 | 93 | 103 | 0.85 | 107 | 111 | 0.34 | 138 | 167 | 2.45 | 160 | 180 | 1.69 |
| Age, years |  |  |  |  |  |  |  |  |  |  |  |  |  |  |
| <35 | 26 | 2 | 4 | 3 | 3.85 | 4 | 3 | 3.85 | 4 | 3 | 3.85 | 4 | 4 | 0 |
| 35-49 | 252 | 21 | 19 | 23 | 1.59 | 22 | 25 | 1.19 | 20 | 26 | 2.38 | 24 | 28 | 1.59 |
| 50-64 | 437 | 37 | 34 | 37 | 0.69 | 42 | 41 | 0.23 | 40 | 48 | 1.83 | 49 | 52 | 0.69 |
| 65-74 | 284 | 24 | 20 | 24 | 1.41 | 22 | 26 | 1.41 | 32 | 43 | 3.87 | 38 | 46 | 2.82 |
| 75+ | 183 | 15 | 16 | 14 | 1.09 | 17 | 15 | 1.09 | 42 | 47 | 2.73 | 45 | 50 | 2.73 |
| Grade |  |  |  |  |  |  |  |  |  |  |  |  |  |  |
| I | 141 | 12 | 4 | 3 | 0.71 | 5 | 3 | 1.42 | 12 | 11 | 0.71 | 14 | 12 | 1.42 |
| II | 500 | 42 | 32 | 24 | 1.6 | 38 | 27 | 2.2 | 54 | 57 | 0.6 | 65 | 61 | 0.8 |
| III | 541 | 46 | 57 | 77 | 3.7 | 64 | 81 | 3.14 | 72 | 99 | 4.99 | 81 | 106 | 4.62 |
| ER Status |  |  |  |  |  |  |  |  |  |  |  |  |  |  |
| Negative | 314 | 27 | 39 | 61 | 7.01 | 44 | 65 | 6.69 | 48 | 72 | 7.64 | 55 | 77 | 7.01 |
| Positive | 868 | 73 | 54 | 41 | 1.5 | 63 | 46 | 1.96 | 90 | 95 | 0.58 | 105 | 103 | 0.23 |

Table S4. Beta coefficients and standard errors for prognostic factors included in the constrained Eastern Cancer Registration and Information Centre (ECRIC) breast cancer prognostic models

|  | **ER Positive Model** | | **ER Negative Model** | |
| --- | --- | --- | --- | --- |
| **Prognostic Factor** | **Coefficient** | **SE** | **Coefficient** | **SE** |
| Number Positive Nodes*  (0, 1, 2-4, 5-9, 10+) | 0.56 | 0.07 | 0.44 | 0.07 |
| Tumour Size, mm*  (<10, 10-19, 20-29, 30-49, 50+) | 0.36 | 0.07 | 0.37 | 0.09 |
| Tumour Grade*  (Low, Intermediate, High) | 0.84 | 0.20 | 0.35 | 0.18 |
| Detection by Screening | -0.35 | 0.10 | -0.15 | 0.19 |
| Chemotherapy | -0.33 | 0.07 | -0.25 | 0.10 |
| Hormone therapy | constrained to  overview  estimate |  | set to zero |  |

* modelled as ordinal continuous

Table S5:

Baseline survival for breast cancer mortality by Oestrogen Receptor (ER) status and competing mortality

| Year | ER positive | ER negative | Competing mortality |
| --- | --- | --- | --- |
| 1 | 0.9999 | 0.9982 | 0.9995 |
| 2 | 0.9996 | 0.9921 | 0.9985 |
| 3 | 0.9990 | 0.9862 | 0.9974 |
| 4 | 0.9982 | 0.9817 | 0.9964 |
| 5 | 0.9974 | 0.9773 | 0.9954 |
| 6 | 0.9967 | 0.9739 | 0.9944 |
| 7 | 0.9959 | 0.9724 | 0.9933 |
| 8 | 0.9947 | 0.9708 | 0.9923 |
